# Existential distress in advanced cancer: study protocol of a pragmatic randomized controlled trial of a short-term psychodynamic therapy (ORPHYS) compared to usual psycho-oncological treatment (TAU)

**DOI:** 10.1186/s13063-026-09744-x

**Published:** 2026-05-07

**Authors:** Rebecca Philipp, Charlotte Walbaum, Carsten Bokemeyer, Ulrike Dinger, Martin Härter, Barbara Hemsen, Steffen Holsteg, André Karger, Uwe Koch, Levente Kriston, Susanne Lezius, Reinhard Lindner, Imad Maatouk, Karin Oechsle, Isabelle Scholl, Anna Wagner, Sigrun Vehling

**Affiliations:** 1 Department of Medical Psychology, University Medical Center Hamburg-Eppendorf, Martinistr. 52–W26, Hamburg, Germany; 2 Department of Oncology, Hematology, and Bone Marrow Transplantation with section of Pneumology, University Medical Center Hamburg-Eppendorf, Martinistr. 52–O24, Hamburg, Germany; 3 Clinical Institute of Psychosomatic Medicine and Psychotherapy, Medical Faculty & University Hospital Düsseldorf, Heinrich-Heine University, Moorenstr. 5, Düsseldorf, Germany; 4 Department of Medical Biometry and Epidemiology, University Medical Center Hamburg-Eppendorf, Martinistr. 52–CPW 1, Hamburg, Germany; 5 Institute of Social Work, University of Kassel, Arnold-Bode-Strasse 10, Kassel, Germany; 6 Department of Internal Medicine II, Chair of Integrated Psychosomatic Medicine and Psychotherapy, University Hospital Würzburg, Oberdürrbacher Str. 6, Würzburg, Germany

**Keywords:** Cancer, Psycho-oncology, RCT, Existential distress, Psychodynamic psychotherapy, End-of-life

## Abstract

**Background:**

As improvements in anti-cancer treatments have extended survival, patients with advanced cancer and their family caregivers face existential tension between engaging in life and coping with uncertainty about illness trajectory and the course of treatment. For a subgroup, this tension is associated with overwhelming fear and existential distress. Such adjustment difficulties may increase the risk of mental disorders, poor quality of life, and suicidality, and impair prognostic awareness and patient-clinician communication. Despite growing interest in open conversations about end-of-life issues, systematic evidence on effective psychotherapies to best support psychological adaptation in patients with high levels of existential distress is still scarce. We aim to evaluate the effectiveness of a short-term psychodynamic therapy (ORPHYS) to mitigate existential distress compared to usual psycho-oncological treatment (TAU).

**Methods:**

We conduct a two-arm parallel randomized controlled trial with an active control group. ORPHYS is a manualized individual face-to-face psychotherapy focusing on emotional and relational conflicts specific to cancer patients’ illness situation. Treatment lasts between 5 and 11 months with 15 to 31 weekly sessions (50 min). TAU includes at least one individual session provided by physicians or psychologists with experience in psycho-oncological care. Patients will be assessed pre-intervention and 3, 6, 9, and 12 months after baseline. Target sample size is 160 randomized participants. We recruit patients with stage III/IV solid tumors or advanced hematological cancer and clinically significant existential distress from psycho-oncology clinics and referring oncologists at Hamburg, Düsseldorf, and Würzburg Comprehensive Cancer Centers, Germany. The primary outcome is demoralization (Demoralization Scale-II). Secondary outcomes include diagnoses of affective, anxiety and stress-related disorders, death anxiety, dignity-related distress, and quality of life. Outcome assessments are conducted via self-report questionnaires and diagnostic interviews. Linear mixed models examine outcome differences between trial arms. A confirmatory test of the group contrast at 6-month follow-up after baseline is conducted.

**Discussion:**

Due to an aging population and prolonged survival, there is a growing demand to help patients deal with existential challenges undergoing palliative cancer care. The study will contribute to knowledge about how clinicians can best help patients with advanced cancer who substantially struggle with uncertainty at the end of life.

**Trial registration:**

German Clinical Trials Registry, DRKS00038173. Registered October 20th, 2025, https://drks.de/search/en/trial/DRKS00038173.

ClinicalTrials.gov, NCT07312760. Registered December 30, 2025, https://clinicaltrials.gov/study/NCT07312760.

**Supplementary Information:**

The online version contains supplementary material available at 10.1186/s13063-026-09744-x.

## Introduction

### Background and rationale {9a}

#### Background

Advances in anti-cancer treatments have led to significant changes for patients with incurable cancer and their family caregivers. Along with extended survival, many patients experience an illness trajectory with major temporary improvements [1]. Still, patients often feel emotionally overwhelmed by the need to cope with the existential tension between engaging in life and the uncertainty about response to treatment, potential progress, and physical deterioration. Patients and their family caregivers thus face the challenge of coping with conflicting emotional states which may detrimentally impact existing relationship dynamics. For example, advanced cancer can dramatically increase patients’ dependency needs and lead to intra- and interpersonal conflicts regarding the wish to be cared for versus fear of loss of control [2, 3].

For patients, this challenge can exceed resources for psychological adjustment and result in symptoms of demoralization (e.g., subjective sense of “not knowing how to go on”; [4]) and death anxiety (e.g., fears about the impact of one’s death, not having enough time, prolonged suffering; [5]). Both constructs, among others (e.g., dignity-related distress; [6]), can be subsumed under the concept of existential distress [7]. Cancer patients with pre-existing mental health problems may be more vulnerable to such distress. Existential distress may range in severity from transient adjustment difficulties to severe, ongoing despair [8]. Research shows that approximately every second patient with advanced cancer experiences significant existential distress [9]. Every third patient shows psychological difficulties that qualify for a mental disorder, including adjustment disorder, major depression and anxiety disorders [10, 11]—a prevalence two- to fourfold higher compared to the general population [12]. In addition to the increased risk for mental disorders, patients with high existential distress are more likely to experience lower quality of life, suicidality, impaired patient-clinician communication, limited prognostic awareness, and other adverse health care outcomes [2, 9, 13].

Current practice guidelines suggest open and early conversation about end-of-life issues in oncological, palliative care, as well as psychotherapeutic settings [14]. Evaluations of specific approaches on how such conversations can effectively reduce existential distress are rare [15, 16]. According to previous research, these conversations are especially challenging for cancer patients with high existential distress and pre-existing mental health problems—a subgroup of about 20% across advanced cancer clinical settings [9]. So far, this subgroup may have been underrepresented in psychotherapeutic interventions.

Evidence on psychotherapies addressing the subjective psychosocial needs of cancer patients shows that most trials used broad inclusion criteria to facilitate intervention access, provided 2 to 8 sessions, and used predominantly supportive techniques [17, 18]. In a meta-analysis, the types of psychotherapies applied in various serious illness and palliative care settings included traditional and third-wave cognitive-behavioral interventions (*n* = 21/36), existential approaches (*n* = 12/36), and interpersonal therapy (*n* = 3/36) [19]. When compared to no psychosocial support, manualized approaches including Managing Cancer and Living Meaningfully therapy, meaning-centered psychotherapy, dignity therapy, and supportive-expressive group therapy found small to moderate effects on reducing depressive symptoms and improving quality of life in advanced cancer populations [20–25]. Fewer studies investigated reduction in demoralization symptoms and showed limited effects [26]. This may be due to subgroups of cancer patients with high levels of death anxiety, who may feel too overwhelmed to engage in end-of-life conversations and thus cannot benefit from the provided psychosocial or psychotherapeutic support [21]. Jackson and Emanuel [2] propose that symptoms of existential distress can be alleviated if their subjective meaning for each patient can unfold over time. This may be harder to achieve in short-term therapies focusing on stabilization.

#### Rationale

Psychodynamic psychotherapy provides a sound theoretic framework on how the diagnosis of a life-threatening illness may reactivate emotional conflicts, which then contribute to impaired psychological adjustment [27–29]. Depending on patients’ early relational experiences, fears of physical suffering or death from illness may translate into fears of dependency or abandonment while at the same time feeling a strong need for intimate care. Such emotional conflicts may result in severe existential distress, e.g., demoralization. Studies support the idea that balancing such conflicting inner realities is a central psychological challenge for patients [30–33]. Recent literature on psychodynamic psychotherapies suggests that seriously ill patients can be supported in dealing with existential and psychological symptoms caused by the illness by cautiously addressing dysfunctional coping mechanisms (rigid defense mechanisms such as denial, rationalization, affect isolation) and unconscious emotional conflicts in a supportive and holding environment [34]. Yet, systematic evidence on the effectiveness of psychodynamic psychotherapies in advanced cancer patients is limited [35].

We thus developed ORPHYS (short-term psychodynamic psychotherapy in serious physical illness, [36]), a short-term psychodynamic psychotherapy, which was found feasible and acceptable to the target population in a pilot study [ClinicalTrials.gov NCT05520281]. ORPHYS aims to alleviate symptoms of existential distress in seriously ill patients by exploring reactivated emotional conflicts and thus gaining a deeper understanding of the symptoms’ subjective meaning in order to contain and balance potentially overwhelming fears and despair associated with the illness.

### Explanation for the choice of comparator {9b}

We chose an active comparator to test for comparative effectiveness between a new treatment (ORPHYS) and usual psycho-oncological treatment (TAU) to gain systematic evidence on how to best support this population at the end of life.

## Objectives {10}

### Objectives

Our study aims to test the effectiveness of a short-term psychodynamic therapy (ORPHYS) compared to usual psycho-oncological treatment (TAU) to reduce existential distress in patients with advanced cancer in a pragmatic randomized controlled trial. Compared to usual psycho-oncological treatment, we expect patients who receive ORPHYS to report significantly lower demoralization (primary outcome, assessed by self-report measure Demoralization Scale-II (DS-II, [37])), fewer diagnoses of mental disorders, lower death anxiety, lower dignity-related distress, and higher quality of life (secondary outcomes, assessed by structured diagnostic interview for mental disorders and self-report questionnaires) at the end of treatment. We further aim to explore the potentially beneficial effect of ORPHYS on prognostic awareness and perception of end-of-life discussions, the perceived relationship with health care providers, individual adaptation at the end of life, intensity of medical treatment at the end of life as well as experiences and individual adaptation of patients’ family caregivers (assessed by self-report measures, semi-structured interviews, and medical chart review).

## Methods: Patient and public involvement, and trial design

### Patient and public involvement {11}

Participants were involved in the development of the ORPHYS intervention and design of patient-centered assessments. Focus interviews with volunteering patients who had participated in a previous cohort study and reported a high need for support were conducted. Interviews assessed patients’ perspectives and needs regarding intervention content and focus, experience with assessment of existential distress, and missing aspects in assessments. Participants will be included in this study irrespective of their gender or ethnic background. Based on the results from the pilot study [ClinicalTrials.gov NCT05520281, [38]], ORPHYS is feasible and acceptable to the target population. There are no further PPI activities planned for this trial.

### Trial design {12}

We will conduct a two-arm, parallel, pragmatic randomized controlled trial with an active control group and 5 points of assessment (pre-intervention [baseline], 3, 6, 9, and 12 months after baseline). Patients in the intervention group will receive a manualized short-term psychodynamic psychotherapy (ORPHYS), while patients in the control group will receive usual psycho-oncological treatment (TAU). Primary and secondary outcome measures will be assessed using diagnostic interviews (baseline, 6 months and 12 months after baseline) as well as self-report questionnaires, and electronic medical patient records (baseline, 3, 6, 9, and 12 months after baseline). Block randomization with an allocation ratio of 1:1 is carried out using electronically generated numbers.

## Methods: Participants, interventions, and outcomes

### Trial setting {13}

Patients will be recruited via psycho-oncological clinics, referring oncologists, and palliative care specialists and at the University Cancer Centers in Hamburg, Düsseldorf, and Würzburg. They are either patients who seek psychosocial support through the study centers’ psycho-oncology clinics or patients who are referred to the study.

Study personnel will contact patients’ family caregivers via telephone for participation upon patients’ consent. Study personnel will give comprehensive study information and if family caregivers verbally consent to participate, they will be sent written informed consent and study information via mail.

### Characteristics of the people who are needed for the trial

#### Eligibility criteria for participants {14a}

We will include adult patients (a) who were diagnosed with a solid tumor (UICC stage III or IV) or advanced hematological cancer; (b) whose physical condition allows for weekly appointments (including online or home visits) at the start of the treatment; (c) who report existential distress due to at least one of the following concerns: hopelessness, demoralization, current or future death wishes, fear of death and dying, grief, sense of isolation; (d) who report clinically significant distress or impairment in social, occupational, or other important areas of functioning as is required for diagnosis of a mental disorder. Table 1 shows the expected demographic and medical characteristics of patients and familycaregivers participating in the trial.

**Table 1:** Expected characteristics of the people who are needed for the trial

Characteristics	Patients (ORPHYS, TAU)	Family caregivers
*N*	160	50
Mean age	55–65	55–65
Sex	50% female	70% female
Race, ethnicity, and ancestry	Mostly white	Mostly white
Socioeconomic status	30% post-secondary education	25% post-secondary education
Geographic location	60% of patients at study sites are living in urban areas	60% of patients at study sites are living in urban areas
Primary tumor location		NA
Lung	25%	
Prostate	14%	
Breast	15%	
Gynecologic	10%	
Gastrointestinal	25%	
Brain	3%	
Head and neck	3%	
Sarcoma/mesothelioma	2%	
CUP/other	3%	

Data are based on a pilot study [38] and a cohort study [9, 39] investigating this population.

Exclusion criteria are (a) acute suicidality with a specific plan to act; (b) diagnosis of a substance dependence, substance abuse, or psychotic disorder; (c) inability to attend regular appointments in an outpatient setting (e.g., poor physical condition); (d) other current psychotherapeutic or psycho-oncological treatment according to our TAU definition (see below); (e) insufficient German language proficiency to give informed consent or to complete assessments.

### Eligibility criteria for sites and those delivering interventions {14b}

ORPHYS will be provided by licensed psychodynamic psychotherapists or psychodynamic psychotherapists in advanced training. Across study sites, 20 study therapists (physicians, psychologists) received an 8-h ORPHYS training. The training conveys psychodynamic considerations specific to advanced cancer patients (frequent emotional conflicts, destabilization of typical coping patterns, and development of existential symptoms), treatment technique (therapeutic stance promoting positive transference, addressing physical symptoms, transference dynamics and conflicting relational patterns frequently reported for this population) [34], and works with case studies. Study therapists are supervised at a ratio of 1:4 throughout the entire study period.

TAU will be provided by physicians or master-level psychologists with experience in psycho-oncological care. TAU can be provided via the outpatient service of the study center. Patients can also be referred to external care providers in accordance with the routine procedures at the respective study center.

### Who will take informed consent? {32a}

Under the supervision of a licensed psychotherapist or senior physician, experienced trained study personnel will initially screen patients for inclusion criteria via in-person assessment. Trained study personnel with at least advanced psychotherapeutic training will conduct a comprehensive recruitment interview and hand out written standardized patient information of both treatment arms. After obtaining written informed consent, included patients will complete the baseline assessment including self-report measures and a diagnostic interview. Subsequently, included patients will be randomized.

### Additional consent provisions for collection and use of participant data and biological specimens {32b}

If participants discontinue the trial or intervention, we document their reasons (e.g., study or treatment dropout due to an adverse treatment reaction) and assess consent or withdrawal from further assessments.

## Intervention and comparator

### Intervention and comparator description {15a}

#### ORPHYS (intervention group)

ORPHYS (ShORt-Term Psychodynamic Psychotherapy for Serious PHYSical Illness) is a manualized individual face-to-face psychotherapy that was specifically designed for patients with serious physical illness to reduce existential distress [36]. A cancer diagnosis has the potential to reactivate patients’ latent emotional conflicts (e.g., increased need for proximity and dependency vs. fears of abandonment and loss of control), which may elicit clinically relevant existential distress or mental disorders [9, 30, 40]. ORPHYS aims to address occurring psychological symptoms in an overall supportive treatment setting that allows for exploration of these emotional conflicts without destabilizing patients’ defense mechanisms (i.e., mental processes to avoid emotional conflict) in a way that would be detrimental to navigating the reality of cancer treatment and increasing physical limitations. Focusing on the significance of transference-countertransference dynamics (i.e., unconscious emotional patterns arising within the therapist-patient relationship) in psychodynamic psychotherapy, ORPHYS encourages therapists to (temporarily) identify with the intense transference that is typical for advanced cancer patients who have regressed (increased dependency associated with earlier stages of psychological development) due to emotional crisis. A continuous reflection of one’s own countertransference may help to understand dependent, dismissive, or even hostile projections (i.e., transference of intolerable emotional states) as patients’ individual expression of their desire to feel connected [34]. Such a therapeutic stance may enable therapists to flexibly provide their patients with a "Hilfs-Ich"^1^ (supportive) or an object, which is prepared to work through unconscious conflicts (conflict-oriented).

A pilot study indicated feasibility and safety of the treatment (ClinicalTrials.gov NCT05520281). Twenty-two out of 25 included patients reported clinically significant demoralization (the presence of 6 demoralization symptoms). In semi-structured interviews post-intervention, patients experienced ORPHYS as helpful to reflect on the individual meaning of their illness and associated physical and psychological symptoms in the weekly sessions with a sympathetic and approachable therapist. Qualitative analysis of the transcribed therapy sessions indicated that most patients were confronted with highly ambivalent emotions emerging from aggravated emotional conflicts (e.g., facing death vs. living a meaningful life; saying goodbye vs. feeling close to loved ones). In semi-structured interviews post-intervention, therapists reported that they found the ORPHYS manual helpful to provide a holding environment for their patients, in which they were more likely to identify with good internal objects and thus more capable to regulate their affective states and face their losses. By containing potentially overwhelming emotions, therapists helped their patients to find words to relieve some of the distress that arose from threatening or confusing affective states (mentalization) [38].

In its four treatment phases, ORPHYS focuses on the specific situation of cancer patients when coping with emotional and relational difficulties in the context of illness-related fears and the reality of a limited life expectancy [36].

1. Diagnostic sessions, establishing a therapeutic relationship: Exploring illness-related fears and emotional conflicts; formulating a preliminary psychodynamic framework, including defense mechanisms and the level of structural integration
2. Selecting a treatment focus: e.g., crisis intervention, structural deficits, conflict-oriented; choosing a treatment goal: e.g., improved mentalization, improved capacity to endure ambivalence
3. Working towards the treatment goal taking into account the chosen focus, medical crises, and physical deterioration
4. End of treatment: e.g., accompanying the initiated grieving process; reflecting on the simultaneity of saying goodbye (to one another, to life); consolidating previous insights

#### Treatment dosage

In ORPHYS, patients receive 15 to 31 weekly therapy sessions lasting 50 min. This number of therapy sessions is in line with the German Psychotherapy Standard for short-term psychotherapy and typical short-term psychotherapies [41]. It includes 3 to 7 diagnostic sessions, and additional 12 to 24 psychotherapy sessions, depending on patients’ individual needs and goals. The number of sessions translates into approximately 5 to 11 months of treatment duration, taking into account expectable interruptions due to physical condition, medical procedures, or rehabilitation measures. Sessions may be provided via video or telephone. ORPHYS treatment is considered complete per-protocol (PP), if patients have received at least 15 sessions.

#### TAU (control group)

In line with our pragmatic study approach, content and frequency of TAU sessions may vary between and within the study sites. Details of TAU treatment will be documented by the study personnel on a standardized form (see section Therapy process outcomes). Patients receive routine psycho-oncological treatment as provided by the study centers. TAU includes supportive psycho-oncological interventions according to the German Psycho-Oncology Guideline. There is no requirement for a minimum number of treatment sessions. The dosage is flexibly adapted to individual cases and often includes one session every 3 to 4 weeks over the course of a year.

### Criteria for discontinuing or modifying allocated intervention/comparator {15b}

Discontinuation of treatment (intervention or comparator) will be classified as study or treatment dropout. Reasons for dropout will be documented (e.g., study or treatment dropout due to physical deterioration, death, or an adverse treatment reaction). In case participants are no longer able to attend face-to-face therapy sessions due to physical deterioration, the treatment can be provided online or via home visits.

### Strategies to improve adherence to intervention/comparator {15c}

To investigate treatment adherence, ORPHYS sessions will be audio-recorded, transcribed, and rated using the Penn Adherence/Competence Scale for Supportive-Expressive Dynamic Psychotherapy [42]. Treatment adherence will further be ensured by providing and documenting the regular supervision sessions.

### Concomitant care permitted or prohibited during the trial {15d}

Patients are excluded from the study if they receive other current psychotherapeutic or psycho-oncological treatment according to our TAU definition.

### Ancillary and post-trial care {34}

If participants need psychological or psychosocial support after the intervention has ended, they may contact the treatment facility or study staff at any time post-intervention.

### Outcomes {16}

#### Outcomes and measurements

Patient assessment is conducted via a 1-h telephone or face-to-face interview (diagnostic interview, semi-structured interview) and via a standardized self-report questionnaire. Completion of one self-report questionnaire takes approximately 30 to 40 min. Table S1 shows a complete list of outcome measures and other assessments. All self-report questionnaires specified below assess patient-reported outcomes for patients in both groups, ORPHYS and TAU. The assessment battery was found feasible and acceptable in the pilot study.

##### Primary outcome

We assess demoralization using the Demoralization Scale-II (DS-II), a 16-item self-report questionnaire measuring feelings of hopelessness and helplessness as well as loss of meaning and purpose [37, 43]. Items are scored on a 3-point Likert-scale from 0 (never) to 2 (often), with a total score ranging from 0 to 32 (Cronbach’s alpha = 0.93) [44]. Higher scores indicate higher demoralization.

##### Secondary outcomes

We assess the presence of affective and anxiety disorders using the Structured Clinical Interview for the Diagnostic and Statistical Manual of Mental Disorders (DSM-5) [45]. We assess the presence of an adjustment disorder according to the International Classification of Diseases-11 (ICD-11) using the Adjustment Disorder Module of the Composite International Diagnostic Interview (CIDI) [46].

We assess death anxiety using the Death and Dying Distress Scale (DADDS), a 15-item self-report questionnaire measuring concerns related to death and dying in patients with advanced cancer [5, 47]. Items are scored on a 6-point Likert-scale from 0 (no distress) to 5 (extreme distress), with a total score ranging from 0 to 75 (Cronbach’s alpha = 0.91). Higher scores indicate higher levels of death anxiety.

We assess dignity-related distress using two self-report measures: the 7-point Sense of Dignity Item (SDI) ranging from 0 (no sense of loss of dignity) to 6 (extreme sense of loss of dignity) [48] and six items from the Patient Dignity Inventory (PDI) [49] assessing physical symptom distress, body image concerns, and loss of autonomy. PDI items are scored on a 5-point Likert-scale from 0 (no problem) to 5 (an overwhelming problem) (Cronbach’s alpha = 0.91) [50]. Higher scores indicate a higher sense of loss of dignity.

We assess quality of life using the subscales Preparation for the end of life and Life completion of the self-report questionnaire Quality of Life at the End of Life-Cancer-Psychosocial (QUAL-EC-P, [51, 52]). The 9 items are scored on a 5-point Likert scale from 0 (not at all) to 4 (completely), with a total score ranging from 0 to 36 (Cronbach’s alpha = 0.77), and subscale scores ranging from 0 to 16 (Preparation for the end of life) and 0 to 20 (Life completion). Higher scores indicate higher quality of life.

##### Exploratory outcomes

We assess prognostic awareness and perception of end-of-life discussions with health care providers using a semi-structured interview based on the study by El-Jawahri [53]. For prognostic awareness, patients are asked about their perceived goals of the cancer treatment as well as what they perceive to be their oncologists’ goals for their treatment. Patients’ answers will be assigned to one or more categories (e.g., cure, life-prolongation, maintaining quality of life, tumor stabilization) [54]. For end-of-life discussions, patients are asked about whether they discussed their wishes regarding medical treatment (e.g., at the end of life) with their oncologist and how they experienced these conversations.

We assess the perceived relationship with health care providers using the corresponding subscale of the QUAL-EC-P [51, 52]. The 5 items are scored on a 5-point Likert-scale from 0 (not at all) to 4 (completely), with a subscale score ranging from 0 to 20 (Cronbach’s alpha = 0.81). Higher scores indicate a stronger subjective relationship with health care providers.

We assess individual adaptation at the end of life by a variety of psychological constructs, which are measured using reliable and valid self-report questionnaires: end-of-life adaptation (Revised Loss Orientation and Life Engagement in Advanced Cancer Scale, LOLES) [31], depression (Patient Health Questionnaire-9, PHQ-9) [55], anxiety (Generalized Anxiety Disorder Questionnaire-7, GAD-7) [56], desire for hastened death (Schedule of Attitudes Toward Hastened Death–Short Form, SAHD-A) [57], suicidal ideation (Beck Scale for Suicide Ideation, BSS) [58]. Detailed descriptions of the self-report questionnaires can be found in a previous publication [59].

We assess medical interventions near the end of life by reviewing patients’ medical charts. Assessment includes start of last systemic anticancer therapy (chemotherapy, immunotherapy, targeted therapy, hormonal therapy); date of last systemic anticancer therapy (chemotherapy, immunotherapy, targeted therapy, hormonal therapy); emergency room visits in the last 30 days of life, intensive care unit and inpatient hospitalization in the last 30 days of life, documentation of power of attorney/living will, documentation of do-not-resuscitate/do-not-intubate order, date of first receipt of in- or outpatient palliative care,^2^ and date and place of death [61].

##### Other variables

We assess adverse events using the Inventory for Assessing Negative Effects of Psychotherapy (INEP), a 21-item self-report questionnaire measuring to what extent patients experience intrapersonal and interpersonal changes as well as changes regarding work, stigmatization, and therapeutic misconduct [62]. Patients are also asked to indicate whether they attribute these changes to the therapy itself. Six items are scored on a 7-point scale from − 3 (worsening) to 3 (improvement); fifteen items are scored on a 4-point Likert-scale from 0 (not at all) to 4 (completely). If patients attributed changes to the therapy itself, frequencies, means, and standard deviations of negative (− 3 to 1), no (0), and positive changes (1 to 3) are calculated ((Cronbach’s alpha = 0.86).

We assess structural deficits using the OPD-Structure Questionnaire (OPD-SFK, [63]), a 12-item self-report questionnaire measuring personality functioning based on the Operationalized Psychodynamic Diagnosis (OPD, [64]). Items reflect the subscales self-perception, shaping contact, and key relationship models. They are scored on a 5-point Likert-scale from 0 (not at all) to 4 (completely), with a total score ranging from 0 to 48 (Cronbach’s alpha = 0.89). Higher scores indicate higher impaired personality functioning.

We assess interpersonal difficulties using the Inventory of Interpersonal Problems (IIP-32), a 32-item self-report questionnaire measuring distress due to interpersonal problems on eight subscales [65, 66]. Items are scored on a 5-point Likert-scale from 0 (not) to 4 (very much), with a total score ranging from 0 to 128 and subscale scores each ranging from 0 to 32 (Cronbach’s alpha = 0.86) [66]. Higher total scores indicate higher distress due to interpersonal problems; higher subscale scores indicate specific dysfunctional relational patterns.

We assess emotional dependence on others using an 18-item version [67] of the Depressive Experiences Questionnaire (DEQ), a self-report questionnaire measuring feelings of helplessness, loneliness, and fear of rejection due to disruption in a relationship [68–70]. Items are scored on a 7-point Likert-scale from 1 (strongly disagree) to 7 (strongly agree), with a total score ranging from 18 to 126. Higher scores indicate higher dependence on others to regulate emotional distress (Cronbach’s alpha = 0.74–0.83) [71].

We assess perceived relationship communication using the Couple Communication Scale (CSS), a 10-item self-report questionnaire based on the Couple Checkup Scale [72, 73] measuring communication, conflict resolution and relationship satisfaction in romantic relationships. Items are scored on a 5-point Likert-scale from 1 (strongly disagree) to 5 (strongly agree), with a total score ranging from 10 to 50 ((Cronbach’s alpha = 0.91). Higher scores indicate better communication in relationships.

We assess patients’ perceived competence in coping with cancer using subscales Coping competence and Adaptability of the self-report questionnaire Patient Competence Questionnaire (PCQ) measuring resource-oriented coping [74]. The 7 items are scored on a 6-point Likert-scale from 0 (not at all) to 5 (completely), with a total score ranging from 0 to 35, and subscale scores ranging from 0 to 20 (Coping competence, Cronbach’s alpha = 0.74) and 0 to 15 (Adaptability, Cronbach’s alpha = 0.75). Higher scores indicate higher levels of resource-oriented coping.

We collect sociodemographic characteristics (e.g., age, gender, relationship status, children, socioeconomic status, religion/spirituality) within the standardized self-report questionnaire. We collect patients’ medical information (e.g., tumor diagnosis, oncological treatment) via medical chart review.

We assess physical symptom burden using the Memorial Symptom Assessment Scale (MSAS-SF), a 28-item self-report questionnaire measuring the frequency and the distress of physical symptoms due to cancer or its treatment [75]. Items are scored on a 5-point Likert-scale ranging from 0 (not at all distressed) to 4 (very much distressed). The average symptom score is calculated (Cronbach’s alpha = 0.87). Higher scores indicate higher physical symptom burden.

##### Outcomes reported by family caregivers

We assess family caregivers’ experiences with the patient undergoing treatment and their individual adaptation using parallel interviews and caregiver-reported outcomes. Identical assessments for family caregivers include the following:

- ◦ Self-report questionnaires for demoralization (DS-II, [37]), depression (PHQ-9, [55]), anxiety (GAD-7, [56]), and suicidal ideation (BSS, [58]), structural deficits (OPD-SFK, [63]), interpersonal difficulties (IIP-32, [62]), emotional dependence (DEQ, [67]), perceived relationship communication (CCS, [73])
- ◦ Diagnostic interview for affective, anxiety, and adjustment disorders (SCID-5, [45]; CIDI, [76])

Complementary assessments for family caregivers include the following: [73].

- ◦ Sociodemographic characteristics (e.g., age, gender, relationship status, children, socioeconomic status, religion/spirituality, degree of kinship/relationship with patient)
- ◦ Semi-structured interviews for prognostic awareness and end-of-life discussions from the family caregiver’s perspective, for their subjective experience with their relative’s illness, as well as the family caregiver’s individual psycho-oncological treatments (frequency, detailed description of treatment) and use of psychosocial support
- ◦ Self-report questionnaires for death anxiety among family caregivers (Death and Dying Distress Scale-Caregivers, DADDS-CG [77]) and a caregiver version of the LOLES [31]. The latter instrument measures the extent to which the caregiver’s life is influenced by inner conflict regarding their relative’s illness. Its 20 items are scored on a 5-point Likert-scale from 0 (not at all) to 4 (completely) with a total score of 80. Higher scores indicate higher distress due to inner conflict.

Specific psychological constructs for family caregivers are assessed using reliable and valid self-report questionnaires: Anticipatory grief (Marwit–Meuser Caregiver Grief Inventory, MM-CGI-SF, [78]), caregiver guilt (Caregiver Guilt Questionnaire, CGQ, [79]), complicated grief after the patient’s death (Inventory of Complicated Grief, ICG, [80]), the patient's quality of dying and death from the caregivers’ perspective (Quality of Dying and Death Questionnaire, QODD, [81]). Detailed descriptions of these questionnaires can be found in a previous publication [59]. We also assess family caregivers' health-related quality of life using the Short-Form Health Survey (SF-8, [82]), an 8-item self-report questionnaire measuring 8 aspects of physical and mental health-related quality of life. Items are scored on a 6-point Likert scale from 0 (not at all) to 5 (very much). Norm-based scores are calculated [83], higher scores are associated with higher health-related quality of life.

##### Therapy process outcomes

We assess therapeutic alliance using the Working Alliance Inventory–Short Revised (WAI-SR), a 12-item self-report questionnaire measuring goal-orientation of the therapeutic working relationship [84, 85]. Items are scored on a 5-point Likert-scale from 1 (rarely) to 5 (always), with a total score ranging from 12 to 60 (Cronbach’s alpha = 0.90) Higher scores indicate a stronger therapeutic alliance with regard to goal-orientation.

We assess psycho-oncological intervention techniques using an adapted version of the Multitheoretical List of Therapeutic Interventions-30 (MULTI-30), a 30-item self-report questionnaire measuring therapeutic interventions in psychotherapy sessions [86]. We adapted the questionnaire for the use in a psycho-oncological setting by adding 9 items that assess typical contents and interventions in this setting [87]. The 39 items are scored on a 5-point Likert-scale from 1 (not at all typical of the session) to 5 (very typical of the session). Higher scores indicate a more frequent use of intervention techniques.

After each therapy session, ORPHYS patients will complete a short questionnaire including the following assessments: anxiety and depression (PHQ-4, [88]), therapeutic alliance (WAI-SR, [84]), and therapeutic agency (Therapeutic Agency Inventory, TAI, [89]). The TAI is a 15-item instrument measuring patients’ perception of their active participation in the therapeutic process. Items are scored on a 5-point Likert-scale from 1 (not true) to 5 (very true), with a total score ranging from 15 to 75 (Cronbach’s alpha = 0.84). Higher scores indicate a stronger sense of actively influencing therapy and bringing about change. Completion of the self-report questionnaire takes approximately 5 to 10 min.

After each session, ORPHYS therapists will document all therapy sessions on a structured, standardized form, which includes the following information:

- ◦ Date, time of the session, and setting
- ◦ Suicidal thoughts and/or intentions based on a standardized operating procedure [90]
- ◦ Short summary of the session
- ◦ Thoughts on transference-countertransference based on the OPD-3 [89]
- ◦ Psychological or psychiatric symptoms (e.g., mood, concentration, level of energy, level of activity), structural deficits based on the OPD-3 [91]
- ◦ Medical treatment
- ◦ Occurrence and severity of adverse events (AE, new psychological or physical symptoms, progress of disease, hospital admission, death, suicidal thoughts or attempts, difficulties in relationships or other areas of life) rated on a scale from 1 (mild) to 3 (severe), likelihood of an AE being related to the therapy rated on a scale from 1 (none) to 5 (definitely) [92]
- ◦ Dropout (treatment, study, treatment-and-study dropout, or dropout due to adverse event)

Further, ORPHYS therapists will complete the WAI-SR [84].

Trained observers rate audio recordings and transcripts of therapy sessions to assess insight into repetitive dysfunctional relationship patterns and defense mechanisms. Insight is rated on a 5-point Likert-scale from 0 (not at all) to 4 (very much) using the 12-item Insight into Conflictual Relationship Patterns Scale (ICR, [93]). The total score ranges from 0 to 48. Higher scores indicate a more complex understanding of internal states and interpersonal processes. Defense mechanisms are rated by ranking the 150 items of the Q-sort version of the Defense Mechanisms Rating Scale (DMRS-Q, [94]) on an ordinal scale from 1 to 7. Qualitative and quantitative scores will be calculated using the DMRS-Q web-app (https://webapp.dmrs-q.com/).

Dosage and content of TAU treatment will be documented by the study personnel on a standardized form, which includes the following information.

- ◦ Date, time of the session, and setting
- ◦ Short summary of the session
- ◦ Occurrence and severity of adverse events
- ◦ Dropout

A semi-standardized section of the diagnostic interview will assess treatment dosage and frequency as well as utilization of further psychosocial support services (e.g., relaxation training, self-management programs).

##### Treatment adherence

To investigate treatment adherence, ORPHYS sessions will be audio-recorded, transcribed, and rated using the Penn Adherence/Competence Scale for Supportive-Expressive Dynamic Psychotherapy [42]. Treatment adherence will further be ensured by providing and documenting the regular supervision sessions.

### Harms {17}

After each ORPHYS and TAU session, occurrence and severity of adverse events (new psychological or physical symptoms, progress of disease, hospital admission, death, suicidal thoughts or attempts, difficulties in relationships or other areas of life); serious adverse events (death, life-threatening event, prolonged or persistent disability or incapacity); and unanticipated events as well as their likelihood of being related to the treatment will be documented on a structured, standardized form [62, 92]. All adverse, serious adverse, and unanticipated events will be reported in trial publications.

### Participant timeline {18}

Table 2 shows the schedule of enrollment, intervention, and assessments.

**Table 2:**
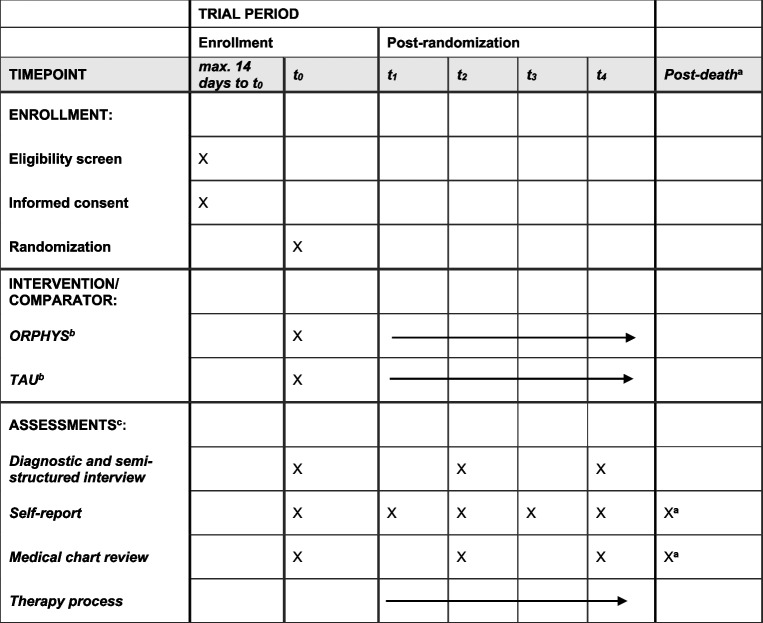
Participant timeline: schedule of enrollment, intervention, and assessments

^a^Assessment is conducted with family caregivers

^b^Arrows indicate continuous delivery of intervention

^c^For a detailed list of assessments see Table S1 in the supporting information

### Sample size {19}

Based on the results for demoralization we found in the pilot study (SD = 5.4), a mean 2-point difference on the Demoralization Scale between ORPHYS and TAU post-intervention is clinically relevant resulting in a medium-sized effect of Cohen’s d = 0.5 [95]. Calculating a two-tailed t-test with a power of 80% (1-β, true positive) and a type I error probability level of alpha = 0.05, 128 patients (64 patients per group) are needed for the post-intervention assessment to detect a moderate effect size of Cohen’s d = 0.5. Assuming a completion rate of 80% (due to patients’ death or physical deterioration) between baseline and post-intervention assessment, 160 patients need to be randomized. Taking into account a participation rate of 70%, 229 patients need to fulfill inclusion criteria.

### Recruitment {20}

We will recruit participants at the study centers in Hamburg, Düsseldorf, and Würzburg. They are either patients who seek psychosocial support through the study centers’ psycho-oncology clinics or patients who are referred to the study. Under the supervision of a licensed psychotherapist or senior physician, experienced trained study personnel will initially screen patients for inclusion criteria via in-person assessment. Trained study personnel with at least advanced psychotherapeutic training will conduct a comprehensive recruitment interview and hand out written standardized patient information on both treatment arms. After obtaining written informed consent, included patients will complete the baseline assessment including self-report measures and a diagnostic interview. Subsequently, included patients will be randomized.

Study personnel will contact patients’ family caregivers via telephone for participation upon patients’ consent. Study personnel will give comprehensive study information and if family caregivers verbally consent to participate, they will be sent written informed consent and study information via mail. In the event of a patient’s death, participating family caregivers will receive another questionnaire 3 months after the patient’s death (post-death survey). Follow-up for deceased patients will be performed until 1 year after inclusion of the last participant.

Diagnostic interviews will be conducted via telephone or face-to-face by trained advanced psychology or medical students; self-report questionnaires and follow-up assessments will be sent via mail. We will document the number of excluded participants as well as reasons for exclusion. For non-responder analyses, we will collect basic demographic and medical data from non-participants upon their consent.

## Assignment of interventions: randomization

### Sequence generation: who will generate the sequence {21a}

The randomization sequence is generated using statistical computing software R, version 4.5.2 [96], and sealed in consecutively numbered envelopes by a person independent of the study personnel.

#### Sequence generation: type of randomization {21b}

Block randomization with an allocation ratio of 1:1 is carried out using electronically generated numbers. Blocks of variable length are used to generate the sequence in order to avoid predictability of allocation. Randomization is stratified by center.

### Allocation concealment mechanism {22}

Study personnel do not have access to the randomization list (concealed allocation sequence). After the included patients provided written informed consent and completed baseline assessment (diagnostic interview, self-report questionnaire), the independent person randomly assigned them to ORPHYS or TAU by opening the envelope and informing a therapist from the respective intervention group.

### Implementation {23}

The therapist then contacts the patient and arranges the session dates. Treatment allocation is communicated either during this conversation or in a separate contact after completion of T0 assessments.

## Assignment of interventions: blinding

### Who will be blinded {24a}

Since this is a psychotherapy study with two active intervention groups, blinding of participants and therapists is not possible. Recruitment interviews and assessments (diagnostic interviews, mailing of self-report questionnaires) are conducted only by trained, clinically experienced research assistants, who have no knowledge of patients’ treatment allocation.

### How will blinding be achieved {24b}

Before each assessment, participants are reminded to not disclose their treatment allocation to study personnel. Study personnel not involved in data collection will perform statistical analysis. Statistical support is provided by the Department of Medical Biometry and Epidemiology at the University Medical Center Hamburg-Eppendorf (SL).

### Procedure for unblinding if needed {24c}

N/a, trial is non-blinded.

## Data collection and management

### Plans for assessment and collection of outcomes {25a}

**Fig. 1:**
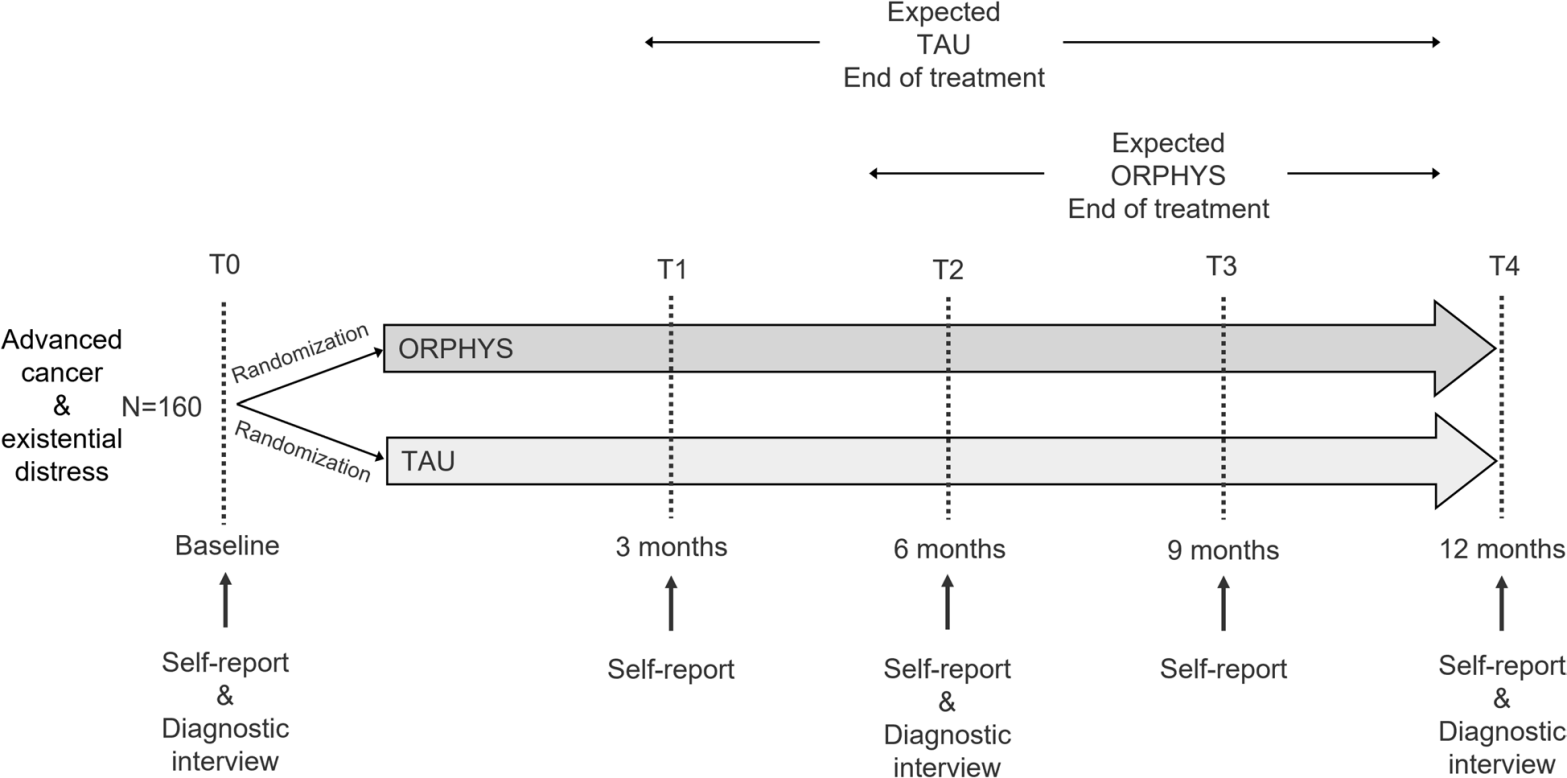
Data collection process

Figure 1 shows the data collection process. After enrollment in the study, participants complete the baseline assessment (diagnostic interview, self-report questionnaire) within 14 days and are randomized afterwards. Post-randomization assessments are collected at fixed intervals (3, 6, 9, and 12 months after baseline assessment). The primary outcome demoralization is measured at baseline, 3, 6, 9, and 12 months after baseline. Based on an expected treatment duration of 24 sessions and a 30% session cancellation rate, we expect treatment to last about 8 months. Taking into account the range of treatment duration from 15 to 31 sessions, we expect completion of treatment after 5 to 11 months. Medical information including end-of-life care will be collected via medical chart review (electronic patient file, medical reports). We will conduct two semi-structured interviews with participating family caregivers at 6 and 12 months. Table S1 shows the planned assessments and corresponding time points.

### Plans to promote participant retention and complete follow-up {25b}

Each ORPHYS and TAU session will be documented on a structured, standardized form including date and time of the session, setting (e.g., face-to-face, telephone, home visit), attendance, occurrence and severity of adverse events, and study or treatment dropout. If patients miss an appointment without canceling in advance, therapists will contact them via phone and document reasons for cancellation and the next planned appointment. If participants do not return the self-report questionnaires within 14 days, study personnel will remind them via telephone.

We document information relevant to the data collection process (e.g., dates of follow-up assessments, return of self-report questionnaires, dropout). If participants discontinue or deviate from intervention protocols, study personnel will document reasons for study or treatment dropout. Outcome data that are collected before dropout will be included in the analyses.

### Data management {26}

We document participants’ personal data (name, contact data), corresponding study IDs, and information relevant to the data collection process (e.g., date of follow-up assessments, return of self-report questionnaires, dropout) in Microsoft Access (LTSC 2021). Throughout the data collection process, participants’ study IDs are used (pseudonymization). After completion of the RCT, the study ID list will be deleted. The Microsoft Access file and the audio recordings of the therapy sessions are stored on encrypted flash drives, which are kept in locked cabinets and backed up once a month. Only senior study personnel have access to the Microsoft Access file and the audio files.

Diagnostic interview sheets and self-report questionnaires are stored in locked cabinets. Research assistants will enter the data of the assessments into IBM SPSS Statistics, Version 29 [97]. The SPSS file is stored on secured servers with regular backup, which can only be accessed by the study personnel. To ensure data quality, 20% of the manually-entered data in Microsoft Access and SPSS will be entered twice and range checks for data values will be performed in SPSS. Inconsistencies will be resolved by checking the original data in the questionnaires.

### Confidentiality {33}

Data will be handled in line with the respective Federal Data Protection Acts. All staff will be required to undergo data management training and sign a data protection commitment document. Data integrity will be monitored closely. Study personnel will undergo Good Clinical Practice training prior to study initiation. Collected data will be archived for 10 years after completion of the study. The data security concept was confirmed with the local data protection officers of each study center.

## Statistical methods

### Statistical methods for primary and secondary outcomes {27a}

To investigate the treatment effect for the primary outcome demoralization at post-intervention, we will calculate a linear mixed model using the difference between post-intervention and baseline scores (change-from-baseline model). The model will include intervention (ORPHYS vs. TAU), time point and the interaction intervention group X time point as fixed effects to test group differences and a random intercept to account for inter-individual differences. The model will include trial site, therapists and patients as nested random effects. We will conduct a confirmatory analysis of the between-group contrast at the 6-month follow-up.

We will investigate treatment effects for the continuous secondary outcomes (death anxiety, dignity-related distress, quality of life) at post-intervention accordingly. To investigate the treatment effect for the binary outcomes (mental disorders) at post-intervention, we will calculate a mixed-effects logistic regression model with intervention (ORPHYS vs. TAU), time point and the interaction intervention group X timepoint as fixed effects as well as trial site, therapists and patients as nested random effects.

Analyses will be performed two-sided with alpha = 0.05. There will be no adjustment for multiple testing. While analysis of the primary outcome will be confirmatory, all other analyses will be exploratory, and specified *p*-values for these analyses should be interpreted descriptively. All analyses will be performed using IBM SPSS Statistics 29 [97] and R, version 4.5.2 [96].

### Who will be included in each analysis {27b}

All analyses are performed in the intention-to-treat (ITT) population.

### How missing data will be handled in the analysis {27c}

Missing values are treated as missing at random and replaced using maximum likelihood estimation within the mixed-model analyses. We will perform the following sensitivity analyses to handle missing data: We will test for systematic study or treatment dropout in both intervention groups regarding the primary outcome at the beginning of treatment as well as age, gender, time since diagnosis and occurrence of illness progression within the last three months (time-dependent covariate). In case of systematic dropout, we will perform sensitivity analyses using multiple imputation to replace missing values due to dropout based on the existing data of similar participants. Because of the seriously ill study population, we expect some dropouts and resulting missing values due to death or physical inability to complete the assessments. We will perform sensitivity analyses by excluding these participants from our analyses.

### Methods for additional analyses (e.g., subgroup analyses) {27d}

#### Sensitivity analyses

We will perform sensitivity analyses by including the covariates age, gender, the time since first diagnosis, and the time since diagnosis of incurable cancer in the linear mixed model based on their association with the primary outcome demoralization in previous studies [9, 13]. Moreover, we will adjust the model for the occurrence of illness progression within the last 3 months by including a binary time-dependent covariate.

### Interim analyses {28b}

We will not perform interim analyses.

### Protocol and statistical analysis plan {5}

We will submit the statistical analysis plan as a supplement under “Study Documents” on ClinicalTrials.gov before data lock.

## Oversight and monitoring

### Composition of the coordinating center and trial steering committee {3d}

Study personnel of the 3 trial sites as well as the Institute of Medical Biometry and Epidemiology are committed to ethical standards and the Guidelines for Safeguarding Good Research Practice by the German Research Foundation [98]. They meet once a month to discuss the recruitment process (e.g., recruitment goals, dropout), data collection and data management (e.g., missing data). Meetings will be organized by the study personnel in Hamburg (coordinating trial site) and led by the principal investigator (SV). ORPHYS therapists participate in group supervision once a month. Supervision will be organized by the principal investigator (SV) and led by two external licensed psychotherapists.

### Composition of the data monitoring committee, its role and reporting structure {28a}

A data-monitoring board, consisting of independent researchers with clinical and statistical expertise as well as expertise in the conduct of related clinical trials, will enable detection of potential adverse events (AE) early on and take appropriate measures due to close monitoring (documentation of therapy sessions, process variables) and supervision, thus minimizing the risk of serious AEs (SAE, suicidality, suicide). If the AE is treatment-related, it is characterized and documented as an adverse treatment reaction (ATR). Serious ATRs (death, life-threatening conditions, need for hospitalization) are reported immediately to the ethics committee. In case of suicidality, therapists are trained to follow a detailed standard operating procedure "suicidality" [88]. They will further contact the principal investigator and responsible local study manager immediately. Study personnel will discuss mild to moderate AEs (e.g., distress during the interviews) with patients and together they will decide on appropriate measures. If participants discontinue the trial or intervention, we document their reasons (e.g., study or treatment dropout due to an ATR) and assess consent or withdrawal from further assessments.

### Frequency and plans for auditing trial conduct {29}

The trial conduct, including participant safety and trial integrity, is monitored by an independent expert, who will audit 10% of all study files every 6 months throughout the course of the study.

### Protocol amendments {31}

Changes to the protocol will be reported in the study registration (https://clinicaltrials.gov/study/NCT07312760).

## Dissemination policy {8}

Dissemination of study results includes the following strategies: publication in peer-reviewed open-access journals, conference presentations, and executive summaries (e.g., for participants, for the funding institution).

## Discussion

The challenge for advanced-cancer patients to psychologically adapt to the profound changes and numerous losses caused by their illness has become a key issue in psycho-oncological research. While every fifth patient suffers from high existential distress and comorbid mental disorders, the number of patients struggling to process the psychological challenges of living with a serious physical illness may increase, as advances in cancer treatments lead to extended survival. Clinicians face difficulties in treating vulnerable patients, who feel overwhelmed by existential fear and avoid engaging in end-of-life conversations that touch emotional conflicts between attachment and loss of shared time, the need to be cared for and loss of autonomy, or self-worth and fears of rejection. We developed ORPHYS in line with current research on psychodynamic interventions and treatment techniques that have been described as helpful in alleviating cancer patients’ existential distress by strengthening patients’ capacity to process intense emotions through establishing a holding and containing therapeutic alliance (ClinicalTrials.gov NCT05520281; [34]).

In this RCT, we address the lack of systematic evidence for the efficacy of psychodynamic psychotherapies in highly distressed and seriously ill cancer patients, who may benefit from regular therapy sessions over a longer period of time to limit the risk of feeling overwhelming despair when touching on existential issues. Compared to previous trials [99, 100], we investigate the effect of psychodynamic psychotherapy on a reduction of symptoms of demoralization, because we hypothesize this outcome to sensitively reflect potential changes due to treatment by taking into account the aim of psychodynamic approaches to increase patients’ capacity to integrate conflicting mental states [101].

We expect that potential methodological limitations will be due to physical deterioration or death in this vulnerable population leading to lower response rates for long-term follow-up assessments, higher treatment dropout rates, and a more challenging acquisition of the pre-specified treatment dosage (e.g., due to interruptions of treatment associated with hospital admissions or rehabilitation).

Results of this RCT will contribute to our knowledge about the benefits of short-term psychodynamic psychotherapy in targeting patients’ existential distress arising from emotional conflicts that are associated with cancer and prolonged survival. Data for the effect of ORPHYS as compared to standard treatment in mitigating patients’ distress will inform clinicians on how to best support patients who struggle with engaging in end-of-life conversations.

## Trial status

This trial is at protocol version 3, dated February 6th, 2026. Date of first enrollment: October 24th, 2025. Recruitment will be completed in December 2027. The study’s clinical trial registration numbers are DRKS00038173 registered with https://www.drks.de/search/de and NCT07312760 registered with https://clinicaltrials.gov/.

## Supplementary Information

Additional file 1. Philipp 2026 supplement trials

## Data Availability

No datasets were generated or analysed during the current study.

## Abbreviations

AE: Adverse event
SAE: Serious adverse event
ATR: Adverse treatment reaction
ITT: Intention-to-treat
ORPHYS: Short-term psychodynamic psychotherapy in serious physical illness
PP: Per-protocol
RCT: Randomized controlled trial
TAU: Usual psycho-oncological treatment
UICC: Union Internationale Contre le Cancer

## Footnotes

1 German for “auxiliary ego,” a treatment technique, in which the therapist supports patients with structural or temporary ego deficits by taking on ego functions.

2 According to German systematiza [60].
