# Supplementary material for: Existential distress in advanced cancer: study protocol of a pragmatic randomized controlled trial of a short-term psychodynamic therapy (ORPHYS) compared to usual psycho-oncological treatment (TAU)
Source: Trials. 2026 May 7;27:358. doi: 10.1186/s13063-026-09744-x (PMC13151140; doi:10.1186/s13063-026-09744-x)
Supplement: Supplementary file 1 — Additional file 1. Philipp 2026 supplement trials [file 13063_2026_9744_MOESM1_ESM.docx]

Table S1. *Patient- and caregiver-reported outcomes, observer-rated assessments.*

|  | **Assessment^a^** | **t0** | **t1** | **t2** | **t3** | **t4** | **d** |
| --- | --- | --- | --- | --- | --- | --- | --- |
| **Primary outcome** | | | | | | | |
| Demoralization (DS-II) | Self-report | X | X | X | X | X |  |
| **Secondary outcomes** | | | | | | | |
| Mental disorders (SCID-5, CIDI) | Diagnostic interview | X |  | X |  | X |  |
| Death Anxiety and Distress Scale (DADDS) | Self-report | X | X | X | X | X |  |
| Dignity-related distress (SDI, PDI) | Self-report | X | X | X | X | X |  |
| Quality of life (QUAL-EC-P) | Self-report | X | X | X | X | X |  |
| **Exploratory outcomes** | | | | | | | |
| Prognostic awareness  End-of-life discussions with health care providers | Semi-structured interview | X |  | X |  | X |  |
| Relationship with healthcare provider (QUAL-EC-P) | Self-report | X | X | X | X | X |  |
| End-of-life adaptation (LOLES) | Self-report | X | X | X | X | X |  |
| Depression (PHQ-9) | Self-report | X | X | X | X | X |  |
| Anxiety (GAD-7) | Self-report | X | X | X | X | X |  |
| Desire for hastened death (SAHD-A) | Self-report | X | X | X | X | X |  |
| Suicidal ideation (BSS) | Self-report | X | X | X | X | X |  |
| Medical treatment at the end of life | Medical chart review |  |  |  |  |  | X |
| **Other outcomes** | | | | | | | |
| Adverse events (INEP) | Self-report |  | X | X | X | X |  |
| Structural deficits (OPD-SFK) | Self-report | X | X | X | X | X |  |
| Interpersonal difficulties (IIP-D) | Self-report | X | X | X | X | X |  |
| Emotional dependence on others (DEQ) | Self-report | X | X | X | X | X |  |
| Perceived relationship communication (CCS) | Self-report | X | X | X | X | X |  |
| Competence in coping with cancer (PCQ) | Self-report | X | X | X | X | X |  |
| Sociodemographic characteristics | Self-report | X |  |  |  |  |  |
| Medical information (e.g., oncological treatment) | Medical chart review | X |  | X |  |  | X |
| Physical symptom burden (MSAS-SF) | Self-report | X | X | X | X | X |  |
| Frequency of psycho-oncological treatments  Utilization of other psychosocial support | Semi-structured interview |  |  | X |  | X |  |
| **Outcomes reported by family caregivers** | | | | | | | |
| Demoralization (DS-II) | Self-report | X | X | X | X | X | X |
| Death anxiety (DADDS-CG) | Self-report | X | X | X | X | X |  |
| End-of-life adaptation (LOLES, caregiver version) | Self-report | X | X | X | X | X |  |
| Depression (PHQ-9) | Self-report | X | X | X | X | X | X |
| Anxiety (GAD-7) | Self-report | X | X | X | X | X | X |
| Suicidal ideation (BSS) | Self-report | X | X | X | X | X | X |
| Health-related quality of life (SF-8) | Self-report | X | X | X | X | X | X |
| Anticipatory grief (MM-CGI-SF) | Self-report | X | X | X | X | X |  |
| Caregiver guilt (CGQ) | Self-report | X | X | X | X | X |  |
| Complicated grief (ICG) | Self-report |  |  |  |  |  | X |
| Patient's quality of dying and death (QODD) | Self-report |  |  |  |  |  | X |
| Structural deficits (OPD-SFK) | Self-report | X | X | X | X | X |  |
| Interpersonal difficulties (IIP-D) | Self-report | X | X | X | X | X |  |
| Emotional dependence on others (DEQ) | Self-report | X | X | X | X | X | X |
| Perceived relationship communication (CCS) | Self-report | X | X | X | X | X |  |
| Sociodemographic characteristics | Self-report | X |  |  |  |  |  |
| Mental disorders (SCID-5, CIDI) | Diagnostic interview | X |  | X |  | X |  |
| Prognostic awareness  End-of-life discussions with health care providers | Semi-structured interview | X |  | X |  | X |  |
| Frequency of psycho-oncological treatments  Utilization of other psychosocial support | Semi-structured interview |  |  | X |  | X |  |
| **Therapy process outcomes** | | | | | | | |
| Therapeutic alliance (WAI-SR) | Self-report |  | X | X | X | X |  |
| Intervention techniques (MULTI-30) | Self-report |  | X | X | X | X |  |
| Therapeutic alliance (WAI-SR)^b^ | Self-report | After each session | | | | | |
| Therapeutic agency (TAI)^b^ | Self-report | After each session | | | | | |
| Anxiety and depression (PHQ-4)^b^ | Self-report | After each session | | | | | |
| Insight into repetitive dysfunctional relationship patterns (ICR)^b^ | Observer-rated | Randomly selected audio-recordings | | | | | |
| Defense mechanisms (DMRS-Q)^b^ | Observer-rated | Randomly selected audio-recordings | | | | | |

^a^ This column corresponds with the type of assessments listed in Figure 1.

^b^ Intervention group only.

*Notes.* t0: baseline assessment, t1: 3-month follow-up, t2: 6-month follow-up, t3: 9-month follow-up, t4: 12-month follow-up, d: caregiver-reported assessment after the patient's death.
